# Non-Alcoholic Fatty Liver Disease (NAFLD) Is an Independent Risk Factor for Developing New-Onset Diabetes After Acute Pancreatitis: A Multicenter Retrospective Cohort Study in Chinese Population

**DOI:** 10.3389/fendo.2022.903731

**Published:** 2022-05-25

**Authors:** Yingqi Lv, Jun Zhang, Ting Yang, Jinfang Sun, Jiaying Hou, Zhiwei Chen, Xuehua Yu, Xuelu Yuan, Xuejia Lu, Ting Xie, Ting Yu, Xianghui Su, Gaifang Liu, Chi Zhang, Ling Li

**Affiliations:** ^1^Department of Endocrinology, Zhongda Hospital, School of Medicine, Southeast University, Nanjing, China; ^2^Key Laboratory of Environmental, Medicine Engineering, Ministry of Education, school of Public Health, Southeast University, Nanjing, China; ^3^Department of Endocrinology, Changji Branch, First Affiliated Hospital of Xinjiang Medical University, Xinjiang, China; ^4^Department of Endocrinology, Hunan Provincial People’s Hospital (First Affiliated Hospital of Hunan Normal University), Changsha, China; ^5^Department of Gastroenterology, Hebei General Hospital, Shijiazhuang, China; ^6^Department of Endocrinology, Yixing Second People’s Hospital, Wuxi, China; ^7^Department of Gastroenterology, Zhongda Hospital, School of Medicine, Southeast University, Nanjing, China

**Keywords:** acute pancreatitis, glucose homeostasis, risk factors, non-alcoholic fatty liver disease, diabetes of the exocrine pancreas

## Abstract

**Background:**

Numerous studies validated frequent glucose dysfunction in patients with acute pancreatitis (AP). However, the prevalence of new-onset diabetes in individuals after a first episode of AP varies widely among previous studies. This study aims to determine the incidence of post-acute pancreatitis diabetes mellitus (PPDM-A) in Chinese people and further identify potential risk factors that influence diabetes development in patients with AP.

**Methods:**

This was a multi-center retrospective cohort study including 6009 inpatients with a first attack of AP. A total of 1804 patients with AP without known endocrine pancreatic disorders or other pancreatic exocrine diseases were eligible for analysis. Data was collected from medical records by hospital information system and telephone follow-ups after discharge. The multiple logistic regression analysis was established to evaluate the potential influencing factors of PPDM-A.

**Results:**

The prevalence of newly diagnosed diabetes after a first episode of AP in China was 6.2%. Data showed that patients who developed PPDM-A were more likely to be younger (X^2^ = 6.329, *P* = 0.012), experienced longer hospital stays (X^2^ = 6.949, *P* = 0.008) and had a higher frequency of overweight or obesity (X^2^ = 11.559, *P* = 0.003) compared to those with normal glycemia. The frequency of stress hyperglycemia on admission (X^2^ = 53.815, *P* < 0.001), hyperlipidemia (X^2^ = 33.594, *P* < 0.001) and non-alcoholic fatty liver disease (NAFLD) (X^2^ = 36.335, *P* < 0.001) were significantly higher among individuals with PPDM-A compared with control group. Also, patients with PPDM-A were more likely to be hyperlipidemic AP (X^2^ = 16.304, *P* = 0.001) and show a higher degree of severity (X^2^ = 7.834, *P* = 0.020) and recurrence rate (X^2^ = 26.908, *P* < 0.001) of AP compared to those without diabetes. In addition, multiple logistic regression analysis indicated that stress hyperglycemia, hyperlipidemia, NAFLD and repeated attacks of AP were the independent influence factors for developing PPDM-A.

**Conclusion:**

Our study first demonstrated the prevalence of secondary diabetes in Chinese patients after AP. The disorder of glucose metabolism in individuals with AP should be regularly evaluated in clinical practice. Further studies are needed to verify the relationship between liver and pancreas in keeping glucose homeostasis under AP condition.

## Introduction

Acute pancreatitis (AP), with a significantly increased incidence, is one of the most common gastrointestinal diseases characterized by a local and systemic inflammatory response and has a complex and variable clinical course which is difficult to evaluate the prognosis at an early stage (1). Although mild AP seems to be self-limiting and usually recover within one week, approximately 20% of patients develop moderate or severe acute pancreatitis, accompanied by pancreatic or peripancreatic necrosis or organ failure, or both, and the mortality range varying between 20% and 40% (2, 3).

The pancreas is an accessory organ of digestion known to have dual functions in the exocrine and endocrine systems, which are closely linked anatomically and physiologically (4).One manifestations of this interplay is the development of diabetes of the exocrine pancreas (DEP), also known as type 3c DM (T3cDM) (5). Recently a retrospective cohort study indicated that DEP, after type 2 diabetes, has become the second most common type of adults-onset diabetes, with a higher prevalence than type 1 diabetes (6). As a core feature of DEP, post-acute pancreatitis diabetes mellitus (PPDM-A) has not attracted much attention in clinical practice so far. A considerable proportion of patients often suffer from a “brittle diabetic state” (great variability in glucose homeostasis and additional risk of hypoglycemia), malabsorption of nutrients and micronutrients due to pancreatic exocrine insufficiency, severe gastrointestinal symptoms (including steatorrhea and flatulence), and muscle atrophy (7, 8). Improper diagnosis and treatment strategies further significantly influence their quality of life.

A growing body of evidence concerning the incidence of PPDM-A is still in dispute. A recently published systematic review and meta-analysis by our workgroup reported that new-onset diabetes occurred in 23% of patients with AP (9), in line with previous research conducted by Das et al (10). However, other studies provided inconsistent prevalence rates (11). In particular, few studies provide insight into the frequency of PPDM-A in Chinese patients (12). In addition, the risk factors associated with PPDM-A progression also remain controversial. Some features including the severity of pancreatitis, etiology, the extent of necrosis, presence of infection and other metabolic indicators were considered to be related with the development of diabetes in some of previous research but made no sense in other studies (11). More evidence is needed to verify these contradictory results and help improving optimal management strategies of this special type of diabetes.

This study aims to establish the prevalence of new-onset diabetes in Chinese adults after AP through medical records collection and telephone follow-ups. Further, we try to analyze the potential risk factors accounting for glucose homeostasis of patients who experienced AP in order to identify susceptible populations and provide reasonable basis for development of management guidelines and recommendations on PPDM-A.

## Research Design and Methods

### Study Design and Ethics Statement

This was a multi-center retrospective cohort study including all inpatients with a first attack of AP from 1 January 2016 to 31 December 2020 at Zhongda Hospital of Southeast University, Nanjing Drum Tower Hospital, Yixing Second People’s Hospital, First Affiliated Hospital of Xinjiang Medical University, Hunan Provincial People’s Hospital and Hebei General Hospital. Written informed consent was obtained from all participants. This study was approved by the ethics committee of all participating institutions. Prior to analysis, all patient information was anonymized and deidentified for privacy.

### Study Population

The cohort eligible for this study comprised all adult individuals (>18 years) experienced a first attack of AP during the study period. In accordance with the revised Atlanta classification, the diagnosis of AP is based on two of the following three criteria (13): (1) a sudden onset of persistent and severe upper abdominal pain, often radiating to the back (2) serum amylase or lipase levels (or both) of at least three times the upper limit of normal, or (3) characteristic imaging findings consistent with AP on contrast-enhanced CT, MRI, or transabdominal ultrasound. The exclusion criteria were as follows: I. not admitted to hospital within 48 hours; II. recurrent or chronic pancreatitis; III. previous history of diabetes or glucose-lowering therapy; IV. abnormal glycated hemoglobin (HbA1c) during hospitalization; V. other pancreatic injury including trauma, pancreatectomy, neoplasm, cystic fibrosis, hemochromatosis, fibrocalculous pancreatopathy or rare genetic disorders; VI. severe cardiac, hepatic or renal insufficiency or malignant diseases; VII. immune system disorders or under hormone treatment; VIII. pregnancy or lactation; IX. data deficiency >10% due to mental illness or other causes failing to the follow-up call.

### Data Collection

This multi-center retrospective cohort study was conducted in Chinese inpatients with first attack of AP who were follow up from the date of admission to the time they developed diabetes or the end of the observation period (31 December 2020). Information on the demographic parameters (gender, age, ABO blood type), hospital stays, medical and family history, severity and etiology of AP, trypsin (amylase and lipase), fasting and random blood glucose, serum calcium, C-reactive protein (CRP), hepatic and renal functions, lipid profiles, infection condition and treatment options were obtained through hospital information system (HIS). During telephone follow-ups after discharge, data including diagnosis of DM, blood glucose levels, glucose-lowering therapy, recurrence of AP, history of cigarette smoking, alcohol consumption, dietary habits and physical activity were recorded.

### Definitions and Classifications

Body mass index (BMI) was calculated as weight (kg) divided by the square of height (m). According to the Guidelines for Prevention and Control of Overweight and Obesity in Chinese adults, overweight was defined as BMI greater than or equal to 24 kg/m^2^, and obesity was defined as BMI greater than or equal to 28 kg/m^2^. The severity of AP was defined as mild, moderately severe or severe according to the revised Atlanta classification (13). Patients were classified as Mild AP in the absence of local or systemic complications and organ failure. Moderately severe AP was defined as the case of local complications or systemic complications, and in the absence of persistent organ failure (<48h). In the case of persistent single or multiple organ failure (>48h), patients were considered severe AP. 2012 International Association of Pancreatology (IAP)/American Pancreatic Association (APA) guidelines recommend identification of the etiology of AP as early as possible (14). Patients were classified as biliary, hyperlipidemic, alcoholic or idiopathic AP, respectively. On the basis of the 2021 American Diabetes Association (ADA) standards, PPDM-A was defined as new-onset diabetes after AP in the absence of a history of pre-existing diabetes before the AP episode (5). In accordance with the ADA consensus definition, stress hyperglycemia was defined in hospital-related hyperglycemia with an admission HbA1c <47.5 mmol/mol(6.5%) and fasting glucose≥7.0 mmol/L or random glucose≥11.1mmol/L without evidence of previous diabetes (15). Hyperlipidemia was defined as hypertriglyceridemia, hypercholesterolemia, or both, on the basis of the Guidelines for Prevention and Treatment of Hyperlipidemia in Chinese adults. Endoscopic retrograde cholangiopancreatography (ERCP) refers to a therapy to extract the stone from choledochal duct by endoscopic sphincterotomy or duodenal papillary balloon dilatation. Surgical therapies included, but not limited to, cholecystectomy, necrectomy, debridement, drainage.

### Statistical Analyses

All analyses were performed in SPSS Statistics (Version 22.0, IBM Corp., Armonk, NY, USA). Continuous variables were presented as means ± standard deviation (SD) or medians [interquartile range (IQR)] in accordance with normality tests, and categorical variables were presented as numbers [percentages (%)]. The independent-samples t-test, Mann-Whitney U test, or chi-square test were used to determine differences between groups. The multiple logistic regression analysis was conducted to analyze the potential risk factors of PPDM-A. All tests were two-sided, and *P*<0.05 was considered to be statistically significant.

## Results

**Figure 1:**
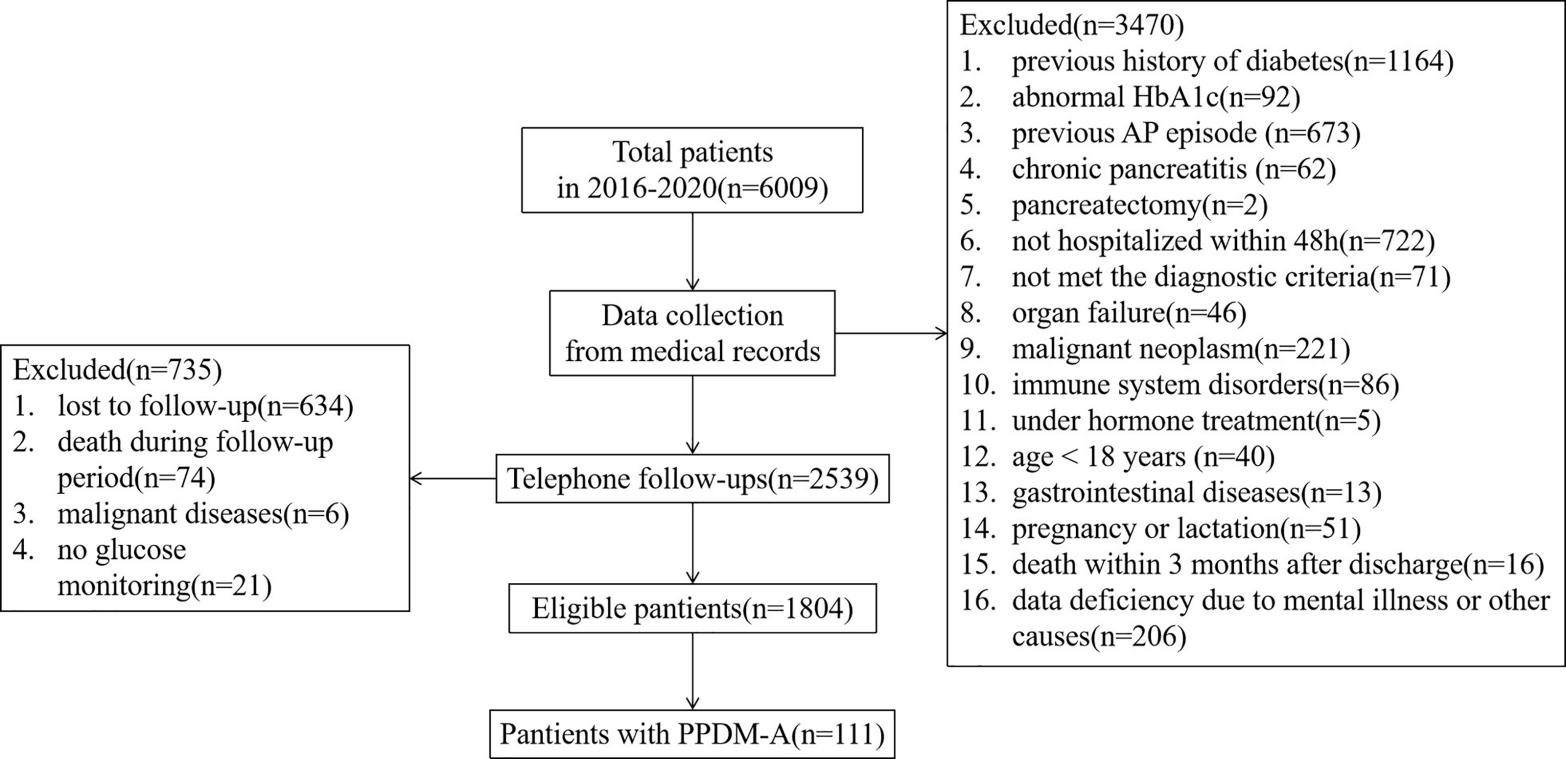
Flow-chart of the study group selection process.

During the period from 2016 to 2020, we identified 6009 individuals with a diagnosis of pancreatitis (**Figure 1**). Among these individuals, 1256 have been diagnosed diabetes previously or had an abnormal HbA1c(≥6.5%) during hospitalization; 737 had experienced recurrent pancreatitis, chronic pancreatitis or other pancreatic injury; 793 were not hospitalized timely (within 48h) or met the diagnostic criteria; 273 had a previous history of severe organ insufficiency or malignant neoplasm; 91 suffered from immune system disorders or long-term hormone treatment; 40 were under 18 years; 19 had a history of mental illness or gastrointestinal diseases; 51 were during pregnancy or lactation; 90 were died in hospital or follow-up period; 221 had incomplete medical records; and 634 were lost to follow-up. After exclusion of above 4205 cases, the final study cohort comprised 1804 eligible new diagnoses of adult-onset pancreatitis with a median follow-up of 3.04(IQR 1.73, 4.47) years.

### Baseline Clinical Characteristics of Patients

Demographic and clinical characteristics of the patients are reported in **Table 1**. Of the 1804 patients finally enrolled, a male predominance was observed (63.1%) and the median age was 48 (IQR 36, 62) years on admission. Patients had a median hospital stays of 10(IQR 7, 14) days and a median BMI of 25.26(IQR 23.03, 27.76) kg/m^2^ at the date of admission. There were 958 patients (53.1%) who were either overweight or obese among all subjects. Cardiovascular disease (CVD) was combined in 165(9.1%) patients and hypertension was diagnosed in 452 (25.1%) patients. 710 (39.4%) patients had a history of biliary tract diseases. There were 492 (27.3%) smokers and 463 (25.7%) drinkers among them. Hyperlipidemia was detected in 731 (40.5%) patients, and 788 (43.7%) patients were accompanied by non-alcoholic fatty liver disease (NAFLD). 1790 patients had fasting or random blood glucose records, and 584 (32.4%) were considered to have stress hyperglycemia. Regarding the etiology of AP, 971 (53.8%) had biliary AP, 240 (13.3%) had hyperlipidemic AP, 68 (3.8%) had alcoholic AP, and 525 (29.1%) had idiopathic AP. As for the severity of AP, 1147(63.6%) were classified as mild AP, 538 (29.8%) as moderately severe AP, and 119 (6.6%) as severe AP. During hospitalization, 1352 (74.9%) patients received conservative medical treatment, 112 (6.2%) underwent ERCP, and 340 (18.8%) underwent surgical operation.

**Table 1:** Patient demographics and clinical characteristics.

Variables	Total	AP without DM	PPDM-A	T/Z/X^2^	P value
N	1804	1693	111	–	–
Age (years)	48 (36,62)	49 (36,63)	47 (36,55)	1.565	0.118
Age (n, %)				6.329	***0.012***
<60years >60years	1309 (72.6%) 495 (27.4%)	1217 (71.9%) 476 (28.1%)	92 (82.9%) 19 (17.1%)		
Gender (n, %)				2.586	0.108
Male Female	1139 (63.1%) 665 (36.9%)	1061 (62.7%) 632 (37.3%)	78 (70.3%) 33 (29.7%)		
Hospital stays (days)	10 (7,14)	9 (7,13)	11 (7,16)	1.733	0.083
Hospital stays (n, %)				6.949	***0.008***
<14days >14days	1415 (78.4%) 389 (21.6%)	1339 (79.1%) 354 (20.9%)	76 (68.5%) 35 (31.5%)		
CVD (n, %)	165 (9.1%)	160 (9.5%)	5 (4.5%)	3.067	0.080
Hypertension (n, %)	452 (25.1%)	421 (24.9%)	31 (27.9%)	0.520	0.471
Biliary tract disease (n, %)	710 (39.4%)	675 (39.9%)	35 (31.5%)	3.035	0.082
Smoking (n, %)	492 (27.3%)	456 (26.9%)	36 (32.4%)	1.588	0.208
Drinking (n, %)	463 (25.7%)	435 (25.7%)	28 (25.2%)	0.012	0.913
SBP (mmHg)	132.8 ± 18.6	132.8 ± 18.8	132.7 ± 16.3	0.047	0.962
DBP (mmHg)	80.9 ± 13.0	80.9 ± 13.0	81.9 ± 12.2	0.858	0.391
BMI (kg/m^2^)	25.26 (23.03,27.76)	25.07 (22.96,27.7)	26.6 (24.09,29.5)	3.264	***0.001***
BMI (n, %)				11.559	***0.003***
<24kg/m^2^ >24kg/m^2^ Unknown	532 (29.5%) 958 (53.1%) 314 (17.4%)	508 (30%) 882 (52.1%) 303 (17.9%)	24 (21.6%) 76 (68.5%)* 11 (9.9%)*		
Amylase (U/L)	512.5 (156,1300)	531.4 (160,1300)	287.7 (118.9,1008.75)	2.582	***0.010***
Lipase (U/L)	339.5 (83,1137.13)	345.5 (83.25,1158.28)	202 (78,883.95)	1.243	0.214
Fast Blood Glucose (mmol/L)	6.4 (5.37,8.0)	6.29 (5.31,7.8)	8.91 (7.0,11.47)	7.761	***<0.001***
Random blood glucose (mmol/L)	7.18 (5.96,8.9)	7.1 (5.9,8.7)	9.88 (7.24,14.06)	7.001	***<0.001***
Stress hyperglycemia (n, %)				53.815	***<0.001***
No Yes Unknown	1206 (66.9%) 584 (32.4%) 14 (0.8%)	1167 (68.9%) 514 (30.4%) 12 (0.7%)	39 (35.1%)* 70 (63.1%)* 2 (1.8%)		
CRP (mg/L)	48.72 (9.9,120.7)	48.72 (9.58,119.65)	48.15 (14.25,177.0)	1.281	0.200
Ca (mmol/L)	2.19 (2.08,2.3)	2.19 (2.08,2.3)	2.2 (2.03,2.30)	0.741	0.459
ALT (U/L)	39 (20,135.9)	40 (20,142)	31 (18.3,72.5)	2.334	0.200
AST (U/L)	34 (19.6,96.25)	34.21 (19.6,98)	27.65 (19,75.03)	1.573	0.116
ALP (U/L)	86 (65.1,130.55)	86 (65.6,131.6)	82 (62.29,111)	1.643	0.101
LDH (U/L)	226 (178.1,321.9)	224 (178,320)	268.06 (186,384.75)	2.292	***0.022***
BUN (mmol/L)	4.5 (3.46,5.9)	4.4 (3.4,5.9)	4.9 (4.0,6.55)	3.273	***0.001***
Cr (umol/L)	66.9 (55,79.4)	66.61 (55,79)	70 (56.36,85.2)	1.622	0.105
UA (umol/L)	302.53 (232,384.6)	300 (230.3,381.75)	332 (269.5,403.85)	2.690	***0.007***
TG (mmol/L)	1.33 (0.82,3.35)	1.27 (0.8,3.1)	3.56 (1.44,8.14)	7.157	***<0.001***
TC (mmol/L)	4.46 (3.64,5.59)	4.42 (3.63,5.52)	5.11 (4.14,7.45)	4.331	***<0.001***
HDL-C (mmol/L)	1.03 (0.8,1.3)	1.04 (0.81,1.3)	0.97 (0.68,1.29)	1.604	0.109
LDL-C (mmol/L)	2.5 (1.91,3.16)	2.49 (1.92,3.16)	2.58 (1.72,3.30)	0.104	0.917
Hyperlipidemia (n,%)				33.594	***<0.001***
No Yes Unknown	1051 (58.3%) 731 (40.5%) 22 (1.2%)	1015 (60%) 657 (38.8%) 21 (1.2%)	36 (32.4%)* 74 (66.7%)* 1 (0.9%)		
Blood type (n, %)				5.573	0.233
A B AB O Unknown	302 (16.7%) 267 (14.8%) 112 (6.2%) 297 (16.5%) 826 (45.8%)	277 (16.4%) 257 (15.2%) 104 (6.1%) 277 (16.4%) 778 (46%)	25 (22.5%) 10 (9.0%) 8 (7.2%) 20 (18%) 48 (43.2%)		
NAFLD (n, %)	788 (43.7%)	709 (41.9%)	79 (71.2%)	36.335	***<0.001***
Infection (n, %)	745 (41.3%)	693 (40.9%)	52 (46.8%)	1.503	0.220
Severity (n, %)				7.834	***0.020***
Mild Moderately severe Severe	1147 (63.6%) 538 (29.8%) 119 (6.6%)	1088 (64.3%) 499 (29.5%) 106 (6.3%)	59 (53.2%)* 39 (35.1%) 13 (11.7%)*		
Etiology (n, %)				16.304	***0.001***
Biliary Hyperlipidemic Alcoholic Idiopathic	971 (53.8%) 240 (13.3%) 68 (3.8%) 525 (29.1%)	927 (54.8%) 213 (12.6%) 65 (3.8%) 488 (28.8%)	44 (39.6%)* 27 (24.3%)* 3 (2.7%) 37 (33.3%)		
Treatment (n, %)				0.003	0.999
Conservatively ERCP Surgery	1352 (74.9%) 112 (6.2%) 340 (18.8%)	1269 (75%) 105 (6.2%) 319 (18.8%)	83 (74.8%) 7 (6.3%) 21 (18.9%)		
Recurrence (n, %)	321 (17.8%)	281 (16.6%)	40 (36%)	26.908	***<0.001***
Dietary habits (n, %) Low-fat High-fat Unknown	1588 (88%) 188 (10.4%) 28 (1.6%)	1496 (88.4%) 178 (10.5%) 19 (1.1%)	92 (82.9%) 10 (9%) 9 (8.1%)	0.070	0.792
Exercise (n, %)				2.410	0.300
Yes No Unknown	1088 (60.3%) 579 (32.1%) 137 (7.6%)	1017 (60.1%) 550 (32.5%) 126 (7.4%)	71 (64%) 29 (26.1%) 11 (9.9%)		

Values are given as means ± standard deviation (SD), median (IQR) or frequencies (percentages). *Statistically significant (P < 0.05) for patients with PPDM-A vs. normoglycemia.

CVD, Cardiovascular disease; BMI, body mass index; SBP, systolic blood pressure; DBP, diastolic blood pressure; CRP, C-reactive protein; ALT, alanine transaminase; AST, aspartate aminotransferase; ALP, alkaline phosphatase; LDH, lactate dehydrogenase; NAFLD, non-alcoholic fatty liver disease; ERCP, Endoscopic retrograde cholangiopancreatography; Cr, creatinine; BUN, blood urea nitrogen; UA, uric acid; TG, triglyceride; TC, total cholesterol; HDL-C, high-density lipoprotein cholesterol; LDL-C, low-density lipoprotein cholesterol; IQR, interquartile range.

The meaning of the bold values is statistical significance (P < 0.05) for patients with PPDM-A vs AP without DM.

### Follow-Up Outcomes and Incidence of PPDM-A

111 patients were defined as developing diabetes after AP, and the overall prevalence of PPDM-A for the entire cohort was 6.2% (111/1804). The patients were divided into two groups according to incidence of diabetes. Of all individuals, 321(17.8%) presented relapse in the follow-up period, and individuals with PPDM-A showed a higher recurrence rate of AP (36.0%) compared with individuals free from diabetes (16.6%) (X^2^ = 26.908, *P* < 0.001). Patients with PPDM-A were more likely to be younger (X^2^ = 6.329, *P=* 0.012) and had a higher frequency of overweight or obesity (X^2^ = 11.559, *P =* 0.003) compared with control group. The frequency of stress hyperglycemia (X^2^ = 53.815, *P* < 0.001), hyperlipidemia (X^2^ = 33.594, *P* < 0.001) and NAFLD (X^2^ = 36.335, *P* < 0.001) were significantly higher among patients with PPDM-A compared to control group. Also, individuals with PPDM-A were more likely to experience longer hospital stays (X^2^ = 6.949, *P*= 0.008), higher lactate dehydrogenase (LDH), blood urea nitrogen (BUN), uric acid (UA), triglyceride (TG) and total cholesterol (TC) levels (all *P* < 0.05), and have a higher degree of severity of AP (X^2^ = 7.834, *P*= 0.020) compared to those without diabetes. In addition, individuals with PPDM-A were more likely to have a higher occurrence of hyperlipidemic AP (X^2^ = 16.304, *P*= 0.001) compared with those with normal glucose levels. However, two groups showed similar distributions of infectious complications, treatments and lifestyle (all *P* > 0.05). The patients’ characteristics and detailed results are listed in **Table 1**.

### Influencing Factors of PPDM-A

The clinical characteristics mentioned above were included into the multiple logistic regression analysis to investigate the potential contributing factors of PPDM-A. Results suggested that stress hyperglycemia, hyperlipidemia, NAFLD, and recurrent pancreatitis were proven to be the independent risk factors for PPDM-A development (see in **Table 2**).

**Table 2:** Factors of PPDM-A by multiple logistic regression analysis.

	β	SE	Wald	*P* value	OR	95% CI, lower	95% CI, upper
Age	-0.208	0.310	0.448	0.503	0.812	0.442	1.492
Gender	-0.117	0.248	0.224	0.636	0.889	0.547	1.446
BMI	-0.287	0.176	2.670	0.102	0.750	0.532	1.059
Hospital stays	0.350	0.252	1.932	0.165	1.419	0.866	2.326
Amylase	<0.001	<0.001	0.509	0.476	1.000	1.000	1.000
Stress hyperglycemia	1.185	0.214	30.770	***<0.001***	3.271	2.152	4.973
LDH	<0.001	<0.001	0.486	0.486	1.000	0.999	1.001
BUN	0.072	0.037	3.819	0.051	1.074	1.000	1.155
UA	<0.001	0.001	0.192	0.661	1.000	0.998	1.002
Hyperlipidemia	0.526	0.258	4.162	***0.041***	1.692	1.021	2.805
NAFLD	0.912	0.247	13.606	***<0.001***	2.489	1.533	4.040
Severity	0.070	0.166	0.175	0.676	1.072	0.774	1.485
Etiology	-0.017	0.090	0.034	0.853	0.983	0.825	1.173
Recurrence	0.907	0.224	16.325	***<0.001***	2.476	1.595	3.844
Constants	-4.470	0.740	36.452	<0.001	0.011	-	-

BMI, body mass index; NAFLD, non-alcoholic fatty liver disease; LDH, lactate dehydrogenase; CI, confidence interval; OR, odds ratio.

The meaning of the bold values is statistical significance (P < 0.05) for patients with PPDM-A vs AP without DM.

## Discussion

Recent evidence has gradually focused on the endocrine insufficiency in patients with pancreatic exocrine diseases, but the pathological mechanism of DEP, especially PPDM-A (one of the biggest contributors to DEP), has not been clarified to date (16). Previous studies revealed that exocrine illnesses could damage pancreatic parenchyma and further impair endocrine function of the pancreas (17). Abnormal blood glucose is a sign of endocrine dysfunction or diabetes occurrence in patients experiencing AP onset. Once progression to permanent hyperglycemia, various diabetes complications will gradually emerge and lower their quality of life. Thus, determining the risk factors regarding glucose metabolism after AP is considered to be helpful to correct hyperglycemia and optimize therapeutic strategy for DEP. In this retrospective cohort study, we investigated the outcomes on recurrence, diabetes and other complications among patients with AP. Data showed that patients who developed PPDM-A were featured by younger age, longer hospital stays, a higher frequency of overweight or obesity, stress hyperglycemia on admission, higher LDH, BUN and UA levels, accompanied by hyperlipidemia and NAFLD, more likely to be hyperlipidemic AP, and had a higher degree of severity and recurrence rate of AP compared to those with normal glycemia. Regression analysis further indicated that stress hyperglycemia, hyperlipidemia, NAFLD and recurrent pancreatitis were the independent influence factors for developing PPDM-A. Stress hyperglycemia in the early phase was crucial to permanent hyperglycemia progression after AP, which might be explained by the insulin resistance induced by endothelial dysfunction and decreased insulin biosynthesis and secretion caused by oxidative stress (18, 19). However, elevated blood glucose after AP attack is difficult to recognize because that the changes are insidious and variable, which may result in the long-term neglect of the glucose monitoring and management in patients with AP and even misclassification as type 2 diabetes.

A meta-analysis reported that approximately 23% (95%CI:16%-31%) of patients with AP developed diabetes (10). In our study, the prevalence of new-onset diabetes in patients with AP was 6.2%, which was lower than the results of the meta-analysis. On the one hand, the difference might be related to the strict exclusion criteria of our study, in which the subjects with a history of recurrent or chronic pancreatitis, pancreatic trauma, surgery, neoplasm, hypoplasia, cystic fibrosis, fibrocalculous pancreatopathy or other pancreatic impairments were excluded. Also, we eliminated those with cardiovascular, cerebrovascular, hepatic, renal or malignant diseases, not promptly hospitalized within 48 hours, previously diagnosed diabetes or received glucose-lowering therapy, abnormal HbA1c during or within 90 days of pancreatitis, immune system disorders or under hormone treatment. According to the diagnostic algorithm to identify PPDM proposed by Petrov MS and Basina M recently, new-onset diabetes after pancreatitis (NODAP) should meet impaired glucose metabolism more than 90 days to rule out stress hyperglycemia which may appear during the course of pancreatitis or within 90 days after hospitalization. Such temporary hyperglycemia may reflect the acute stress reaction or be a response of treatment of pancreatitis such as parenteral nutrition or intravenous infusion of dextrose (20). 24 previous prospective clinical studies included in the above meta-analysis didn’t meet all eligibility criteria in our study. We set such strict exclusion criteria to avoid confounding factors as far as possible and try to approach the real incidence of PPDM-A. In addition, the meta-analysis published by Das et al. only included prospective clinical studies. Since our retrospective study design had inevitable recall bias, we cannot ensure that the prevalence will be similar to what reported by Das et al. even if using similar exclusion criteria. However, we will answer this question in our subsequent prospective studies. In spite of some limitations, it is still the first study about the prevalence of new-onset diabetes in Chinese adults after AP, to the best of our knowledge. On the other hand, regional and racial differences might contribute to inconsistent prevalence rates among studies. Of 145 Finnish patients with severe AP, 43% prevalence of newly diagnosed DM is observed (21). Angelini et al. found that in Italy, the incidence of PPDM-A is 5% among 19 patients with severe AP (22) and 8% among 118 patients with AP (including 83 severe AP and 35 mild AP) (23). A study of 112 patients with severe or mild AP in Turkey showed that the morbidity of DM is 12% (24), and the impaired glucose tolerance was not associated with necrosis or disease severity. Yasuda et al. (25) conducted a prospective cohort study comprising of 41 Japanese patients with severe AP and the development of DM was noted in 39% of individuals. Moreover, multivariate analysis revealed that blood glucose was an independent prognostic factor for the development of DM after severe AP (*P* < 0.05). Another study from India by Gupta et al (26) pointed out that postoperative DM was present in 20% patients with severe AP, and there was no significant correlation of endocrine insufficiency with alcohol intake or gall stone disease (*P*= 0.6), infected pancreatitis (*P*= 0.15) and percent of necrosis (*P*= 0.28). Bharmal et al (27) determined the cumulative incidence of NODAP in New Zealand was 11.2% through 24 months follow-up of 152 patients, and the authors showed that Anthropometrics, pancreatitis-related characteristics, lipid profile, liver enzymes, and markers of inflammation were not significantly associated with the development of high-increasing glycaemia. The exclusion criteria between our study and these studies were similar, although not identical, considering different types of pancreatic injuries, diabetes status and types, follow-up periods, data integrity, survival conditions during hospitalisation and after discharge, and cognitive states. These discoveries were roughly consistent with our results. However, endocrine function after AP was not associated with fatty liver in these studies, probably due to the lack of data on liver assessments and racial differences. Another study of 310 Chinese patients with AP indicated that hyperglycemia on admission, hyperlipidemia, fatty liver, hypertension, ERCP during hospitalization, high levels of LDH and CK, decreased serum calcium, and AB blood type were correlated with elevated blood glucose, and further proved that hyperlipidemia and hyperglycemia on admission were the independent risk factors for diabetes development (12). There may be some potential mechanisms underlying these differences and correlations which needs to be verified. At present, the prevalence of PPDM-A in Chinese population is still uncertain due to insufficient statistical data involving large samples. Our study may offer useful reference to future research.

Glucose homeostasis is important to allow biological processes to proceed normally. In addition to endocrine and exocrine insufficiency of the pancreas itself, several tissues and organs such as liver, gut, brain, muscle and adipose tissue are involved together in the regulation mechanism of glucose homeostasis for patients with DEP. Thereinto, pancreas and liver play key roles in glucose metabolism and thus are regarded as core glucose-controlling target organs (28). As the first messenger of glucose regulation, blood glucose is not only a regulated variable but also a controlled variable, whose level influences the basic homeostatic loop system composed of controllers (pancreas, liver, gut) and effectors (liver, muscle, adipose tissue). In response to an change in blood glucose, the controllers release hormone signals (including insulin, glucagon and incretins), which act on the effectors to produce glucose uptake, transport and utilization, thereby maintaining a stable plasma glucose level.

The effect of exocrine pancreas on endocrine function under physiological conditions can be reflected in the inhibitory effect of amylase and lipase on insulin secretion. One possible explanation is a regulatory mechanism that acts against insulin overproduction and keeps it at a normal level and further avoids exhaustion of β cells (11). In addition to the regulation of insulin secretion, trypsin may also be involved in islet formation and differentiation (29). Inflammation of the pancreas destroys the parenchyma in pathophysiological conditions, and persistent inflammatory response leads to the exocrine pancreatic insufficiency, in which the production of pancreatic enzymes is greatly reduced and thus insulin secretion is released from the inhibitory effect of exocrine enzymes that leads to hyperinsulinemia which has been considered to be responsible for the development of insulin resistance (30). As the extent of pancreatic necrosis increases, the extensive destruction and functional exhaustion of islet β cells led to insufficient insulin secretion. However, the effect of AP severity and extent of necrosis on the risk of developing endocrine insufficiency or secondary hyperglycemia has remained controversial. Historically, PPDM-A develops mostly in those with severe AP and the extent of necrosis was a decisive factor. While this perspective has been challenged by an increasing number of studies. The meta-analysis published by COSMOS group demonstrated that patients with mild AP were at a high risk of developing diabetes and the severity of AP did not materially affect the risk of developing PPDM (10). Several population-based cohort studies also illustrated that there was no correlation between the adjusted risk of PPDM and severity of pancreatitis (31–33). There may be other mechanisms involved in the incidence of PPDM-A besides pancreatic damage per se which remain to be verified by future evidence.

As one of the major metabolic organs, liver plays a critical role in regulating the internal glucose homeostasis. Through glycogenolysis and glycogenesis, liver functions as both a controller and an effector in the homeostatic circuits. Our results showed that patients with AP accompanied by NAFLD were more likely to develop diabetes, highlighting the potential importance of liver in the pathogenesis of DEP. In line with obesity and diabetes, NAFLD is becoming increasingly popular and shares the common pathological mechanisms, i.e. insulin resistance (IR) (34). It has been indicated that immune cells such as macrophages and neutrophils would reside in or infiltrate liver parenchyma under obese conditions and cause chronic inflammation or metabolic disorders that decrease insulin sensitivity thus leading to the development of IR (35). Accumulated adipose tissue releases a large number of inflammatory mediators which can inhibit both the insulin receptor and the action of insulin through various signaling pathways. The adipocytokine interleukin (IL)-6 has been shown to be associated with chronic hyperglycemia and IR after AP (36). Another study showed that obesity-associated adipokine leucine-rich alpha-2-glycoprotein 1 (LRG1) bound with high selectivity to the liver and exacerbated high fat diet-induced hepatosteatosis and IR by increasing *de novo* lipogenesis and suppressing fatty acid β-oxidation (37). LRG1 also inhibited hepatic insulin signaling by downregulating insulin receptor expression. Other pro-inflammatory cytokines such as monocyte chemoattractant protein (MCP)-1 and tumor necrosis factor (TNF)-α were also found to be elevated in patients after AP (38). As it was shown in our study, hyperlipidemia, especially hypertriglyceridemia, significantly increased the risk of PPDM, which could be attributed to inflammation-induced lipolysis. Lipolysis is the hydrolysis of triglycerides to free fatty acids (FFA) and glycerol (39), and FFA, to some extent, was recognized as energy source or for ectopic fat storage in organs such as the liver and pancreas, potentially contributing to IR (40). Hyperlipidaemia-induced pancreatitis has been found to cause diabetes in more patients compared to the other etiologies (41), which was consistent with our results. In the setting of severe hypertriglyceridemia, restricted blood flow and accumulations of FFA could lead to impairment of circulation in capillary beds, ischemic disturbance to the acinar structures, and a resultant increasingly acidic environment, in which the pancreatic lipase could more easily seep out of acinar cells and be activated by FFA to cause additional pancreatic injury (12).

Accordingly, chronic low-grade inflammation and secondary lipolysis contribute to the critical mechanism of IR. When NAFLD continuously progress to chronic inflammation, fibrosis, and cirrhosis, damaged hepatocytes result in the decline in hepatic glycogen synthesis, storage, decomposition, and the ability to regulate blood glucose homeostasis, manifested as impaired islet function and decreased insulin secretion (42). In addition, giving that insulin is mainly metabolized by the liver, the scavenging effect of liver on insulin decreases under pathological conditions, and clinically presenting with hyperinsulinemia, characterized by frequent fasting hypoglycemia and postprandial hyperglycemia. The results of this study reported that patients with PPDM-A were often accompanied by overweight/obesity, hyperlipidemia and NAFLD, suggesting that the hepatopancreatic dialogue may have a potential effect in the development and progression of DEP. Therefore, the intricate information communication between the liver and pancreas seems to be an important autoregulatory mechanism of glucose homeostasis. It is necessary to explore the internal connections to further illustrate the pathogenesis of DEP and provide a new perspective for improving the clinical therapeutic strategy.

The concept of the gut-islet axis, introduced by Unger and Eisentraut in 1969, described the close connection between the gastrointestinal tract and the islets, containing nutrient, neural and hormonal signals from the gut to islet cells(i.e. the gastrointestinal tract could regulate the activity of islet cells by releasing bioactive substances (43). There are dozens of endocrine cells in the gastrointestinal mucosa which secrete a variety of gastrointestinal hormones released into the blood circulation or play a role in regulating insulin secretion and blood glucose homeostasis in the form of local paracrine (44). For example, once the corresponding glucose set point after dietary stimulation was reached, intestinal L cells would initiate glucose regulation at a threshold of about 5.5mmol/L and secrete glucagon-like peptide-1(GLP-1) in response to blood glucose fluctuations, which is an important controller of blood glucose homeostasis (45). Therefore, the crosstalk between the intestine and the pancreas is also an important regulation mechanism of blood glucose homeostasis.

GLP-1 receptor, a seven-transmembrane G protein-coupled receptor, widely distributed in islet cells, cardiomyocytes, liver, kidney, vascular endothelial cells, skin, hypothalamus and other tissues and organs, suggesting that it plays an important extrapancreatic role in addition to the effect of glucose-dependent insulin secretion and glucagon inhibition (46). GLP-1 receptor agonists (GLP-1RA), a new hypoglycemic drug, has been increasingly proved its efficacy and safety in individuals with NAFLD, which can not only reduce hepatic steatosis and inflammation, but improve non-alcoholic steatohepatitis (NASH) (47). A clinical trial involving patients with NASH showed that treatment with semaglutide resulted in a significantly higher percentage of patients with NASH resolution than placebo (48). D-LIFT trial revealed that dulaglutide significantly reduced liver fat content (LFC) and improved γ-glutamyl transpeptidase (GGT) levels in participants with NAFLD (49). These results provide evidence of evidence-based medicine for the close relationship between liver and intestine, indicating that incretin plays an important role in the homeostasis of glucose and lipid metabolism.

Based on our current analysis, people with recurrent AP are at a significantly increased risk for developing PPDM in comparison with those free from repeated attacks of AP, consistent with previous findings from two population-based studies (33) (50). The difference might be ascribed to the pancreas volume variation. A MRI study by the COSMOS group on pancreas volume in individuals after AP demonstrated a significant 22% reduction in total pancreas volume in patients after two or more recurrences of AP whereas no obvious change in those with one or no recurrence (51). It was worth noting that pancreas tail was known to have the highest proportion of the islet of Langerhans, instead of head or body. After two or more recurrences of AP, the pancreas tail was significantly reduced which was directly proportional to the reduction in β-cell mass (52).

Some limitations are present in this study. First, given the discharge glucose values got from telephone follow-ups not blood tests, the retrospective study design consisting of inpatients may suffer from recall bias and thus not be enough to represent the true prevalence of PPDM-A among overall patients in China. Hence, large-scale prospective population-based studies need to be conducted in the future. Second, the pathological features of PPDM-A will be better explained if pancreas islet function can be included in follow-up period, despite it is hard to be realized due to multiple blood collections of insulin or C-peptide releasing test. Third, only 112 patients (6.2%) in the cohort experienced ERCP treatment and 340 patients (18.8%) received surgery, from which we were unable to measure the effects of therapy methods. Thus, whether different treatments of AP could contribute to different consequences regarding the development of hyperglycemia in the long term still needs further verification. Lastly, the mechanisms underlying the association between liver and endocrine pancreas under DEP conditions remain unclear and to be answered by more evidence-based medical researches.

## Conclusion

Our study first determined the prevalence of secondary diabetes in Chinese patients after AP. Stress hyperglycemia, hyperlipidemia, NAFLD and recurrent attacks of AP appear to be the independent risk factors for developing PPDM-A. The disorder of glucose and lipid metabolism in patients with AP should be paid more attention in clinical practice. Furthermore, the mechanisms responsible for the potential relationship between liver and endocrine pancreas need more in-depth studies to explain.

## Data Availability Statement

The raw data supporting the conclusions of this article will be made available by the authors, without undue reservation.

## Ethics Statement

The studies involving human participants were reviewed and approved by the Ethics Committee of Zhongda Hospital of Southeast University. The patients/participants provided their written informed consent to participate in this study. Written informed consent was obtained from the individual(s) for the publication of any potentially identifiable images or data included in this article.

## Author Contributions

YL: Conceptualization, Data curation, and Writing-original draft preparation. JZ: Conceptualization and SPSS Statistics. TYang: Conceptualization and Data curation. JS: Visualization and Investigation. JH, ZC and XHY: Data curation. XLY and XL: Investigation. TX and TYu: SPSS Statistics. XS, GL and CZ: Writing-Reviewing and Editing. LL: Conceptualization, Methodology, and Writing-Reviewing and Editing. All authors contributed to the article and approved the submitted version.

## Funding

This work was supported by National Natural Science Foundation of China (81970717 and 82170845).

## Conflict of Interest

The authors declare that the research was conducted in the absence of any commercial or financial relationships that could be construed as a potential conflict of interest.

## Publisher’s Note

All claims expressed in this article are solely those of the authors and do not necessarily represent those of their affiliated organizations, or those of the publisher, the editors and the reviewers. Any product that may be evaluated in this article, or claim that may be made by its manufacturer, is not guaranteed or endorsed by the publisher.

## Abbreviations

AP, acute pancreatitis; PPDM-A, post-acute pancreatitis diabetes mellitus; DEP, diabetes of the exocrine pancreas; NAFLD, non-alcoholic fatty liver disease; CVD, Cardiovascular disease; NODAP, new-onset diabetes after pancreatitis; IR, insulin resistance; HIS, hospital information system; SBP, systolic blood pressure; DBP, diastolic blood pressure; HbA1c, glycated hemoglobin; BMI, body mass index; CRP, C-reactive protein; ERCP, Endoscopic retrograde cholangiopancreatography; HbA1c, glycated hemoglobin; ALT, alanine transaminase; AST, aspartate aminotransferase; ALP, alkaline phosphatase; LDH, lactate dehydrogenase; Cr, creatinine; BUN, blood urea nitrogen; UA, uric acid; TG, triglyceride; TC, total cholesterol; HDL-C, high-density lipoprotein cholesterol; LDL-C, low-density lipoprotein cholesterol; FFA, free fatty acids; SD, standard deviation; IQR, interquartile range; CI, confidence interval; OR, odds ratio.
